# Overview of *Yersinia pestis* Metallophores: Yersiniabactin and Yersinopine

**DOI:** 10.3390/biology12040598

**Published:** 2023-04-14

**Authors:** Taghrid Chaaban, Yehya Mohsen, Zeinab Ezzeddine, Ghassan Ghssein

**Affiliations:** 1 Nursing Sciences Department, Faculty of Public Health, Islamic University of Lebanon, Khalde P.O. Box 30014, Lebanon; 2 Nursing Sciences Research Chair, Laboratory Educations and Health Practices (LEPS), (EA 3412), UFR SMBH, University Paris 13, Sorbonne Paris Cite, F-93017 Bobigny, France; 3 Department of Medical Laboratory Technology, College of Health and Medical Technologies, Al-Ayen University, Nasiriyah 64001, Iraq; 4 Laboratory Sciences Department, Faculty of Public Health, Islamic University of Lebanon (IUL), Khalde P.O. Box 30014, Lebanon; 5 Faculty of Sciences V, Lebanese University, Nabatieh 1700, Lebanon

**Keywords:** *Yersinia pestis*, pathogenesis, metallophores, metal ions, yersiniabactin, yersinopine

## Abstract

**Simple Summary:**

Although there was a significant decrease in morbidity and mortality due to plague-related infections throughout the 20th century, these have not been eradicated. The plague-causing pathogen is the Gram-negative bacterium *Yersinia pestis*. Several factors cause the virulence of this bacterium including metallophores, which are secondary metabolites for metal ions chelation. *Yersinia pestis* produces two metallophores: yersiniabactin, for iron chelation (siderophore), and an opine type metallophore called yersinopine. This review summarizes all the important characteristics of these two metallophores. Full descriptions of their structures, biosynthesis pathways, and genetic regulation are included in this paper.

**Abstract:**

The pathogenic anaerobic bacteria *Yersinia pestis* (*Y. pestis*), which is well known as the plague causative agent, has the ability to escape or inhibit innate immune system responses, which can result in host death even before the activation of adaptive responses. Bites from infected fleas in nature transmit *Y. pestis* between mammalian hosts causing bubonic plague. It was recognized that a host’s ability to retain iron is essential in fighting invading pathogens. To proliferate during infection, *Y. pestis*, like most bacteria, has various iron transporters that enable it to acquire iron from its hosts. The siderophore-dependent iron transport system was found to be crucial for the pathogenesis of this bacterium. Siderophores are low-molecular-weight metabolites with a high affinity for Fe^3+^. These compounds are produced in the surrounding environment to chelate iron. The siderophore secreted by *Y. pestis* is yersiniabactin (Ybt). Another metallophore produced by this bacterium, yersinopine, is of the opine type and shows similarities with both staphylopine and pseudopaline produced by *Staphylococcus aureus* and *Pseudomonas aeruginosa*, respectively. This paper sheds light on the most important aspects of the two *Y. pestis* metallophores as well as aerobactin a siderophore no longer secreted by this bacterium due to frameshift mutation in its genome.

## 1. Introduction

The Gram-negative pathogen *Yersinia pestis* (*Y. pestis*), which is responsible for causing bubonic, pneumonic, and septicemic plague among humans, emerged from the enteropathogen *Yersinia pseudotuberculosis* [1]. This pathogen is considered a zoonosis because it is carried by rodents worldwide and transmitted within them through a flea vector (*Xenopsylla cheopis*) [2,3]. Effective inter-human transmission may occur through aerosols leading to the development of pneumonic plague [4,5]. However, recent evidence shows that inter-human transmission via aerosol is apparently insignificant [6] and may be due to body lice and fleas [1].

*Y. pestis* evolved multiple factors that enable it to colonize its hosts which include both mammals and insects [7]. An infected flea causes bubonic plague through its bite. Once deposited on the dermis, the bacterium spreads and colonizes the lymph nodes, leading to ‘‘bubo’’ which is the inflammation of the lymph nodes [8].

Historically, three plague pandemics were recorded: The first pandemic, named the Justinian plague after the emperor of the Roman Empire, started in Egypt in 541. The first wave hit the Mediterranean Basin between 541 and 544 [9], then invaded northern Europe and England [10], and was followed by more than 14 waves from 558 to 750/767 [9]. The second wave began at the end of the 1330s in central Asia before spreading to the whole of Western Europe and North Africa, where more than 30% of the European population perished [11]. The third pandemic first appeared in 1772 in Yunnan Province in southwestern China and then spread worldwide via steamships carrying infected rats. Nowadays, it is present in Asia, Africa, and the Americas. Very low mortality rates were noted during this pandemic [11]. The bubonic plague which is transmitted by fleas develops two to ten days after infection with *Y. pestis* [12,13]. Various clinical sample types can be used for the detection of *Y. pestis*, such as bubo aspirates, blood, and respiratory tract samples. For diagnostic tests for pneumonic plague, deep respiratory secretions are needed [14]. In addition, real-time PCR was used to confirm the presence of *Y. pestis* DNA, especially in 2017, during the outbreak of the pneumonic plague in Madagascar [15]. Microbial isolation remains the best available biological diagnostic tests for *Y. pestis* [7].

The plague disease progresses rapidly and the incubation period allows very little time for therapeutic intervention. Indeed, the case fatality rate approaches 100% [5] in a few days if effective antimicrobial treatments are not initiated within 24 to 36 h after the onset of symptoms but is between 25% and 50% when appropriate treatment is administered within 24 hours [14]. Antibiotic treatments should be continued for 10 to 14 days. Enterobacteriaceae including streptomycin, gentamicin, levofloxacin, ciprofloxacin, and chloramphenicol are commonly used and were proven to be effective against plague (CDC 2020). However, antibiotic-resistant cases, particularly streptomycin, were reported [15,16].

The plague is endemic and is still a major public health concern. A total of 26,237 plague cases were reported from 2000 to 2018 in 21 countries across the Americas, Africa, and Asia [7]. The vast majority (97%) of plague cases worldwide were reported in Africa, particularly in the Democratic Republic of Congo and Madagascar. For this reason, current efforts are focused on vaccines against pneumonic plague. Several molecular vaccine candidates were developed, including the V10 vaccine, which is patented by the University of Chicago and provides 100% protection against bubonic and pneumonic plague [17].

Metal ions are vital nutrients needed for the proper biological functioning of living cells. Therefore, bacteria require metal ions for their metabolism, virulence, and transcriptional regulation [18,19].

The infected host, as a part of the immune response, tends to sequester metals to deprive the pathogens of them in a process called nutritional immunity [20,21]. Neutrophils, for instance, respond to infection by releasing metal-binding proteins for restricting bacterial access to metals [22,23]. One of these proteins is calprotectin, which can sequester manganese, iron, and zinc, thus restricting their availability for invading microorganisms [24,25]. It was shown that the sequestration of zinc via calprotectin is a major colonization barrier to a variety of bacteria such as *Salmonella Typhimurium* and *Staphylococcus aureus* [26,27].

Bacteria developed several mechanisms for chelating metal ions from the host- scarce environment to survive nutritional immunity during infection. These mechanisms include three main metal acquisition systems which are: the import of elemental metal ions, extracellular metal sequestering via metallophores, and acquiring metal ions from host proteins [28]. Several bacteria uptake Fe^2+^ by the ferrous transporter Feo, which is essential for virulence and colonization in some bacteria, including *Helicobacter pylori,* the well-known gastrointestinal pathogen [29]. However, Fe^2+^ is not largely available for bacterial uptake due to host restriction, where Fe^3+^ is the predominant iron form in the host [30]. In addition, several bacteria evolved specialized systems for obtaining iron through piracy from nutritional immunity proteins. For example, *Neisseria* spp. and *Haemophilus influenza* have TbpA/TbpB which are TonB-dependent proteins for binding transferrin and extracting Fe^3+^ to transport them into the cells [31]. Other pathogens such as *Streptococcus pneumoniae* and *Treponema pallidum* express lactoferrin receptors [32]. Possessing such systems, bacteria can convert host proteins supposed to restrict their growth into sources of iron. The secretion of metallophores is one of the most powerful mechanisms enabling bacteria to overcome metal ions limitation [33].

Siderophores, which are used for iron sequestering, are considered the most well-known metallophores [34]. These iron-chelating secondary metabolites are biosynthesized within the bacterial cell through the non-ribosomal peptide synthases designated as NRPS or through the NRPS-independent system. After the synthesis step is completed, siderophores are excreted into the extracellular medium to scavenge iron [35]. In Gram-negative bacteria, after complexing iron, the loaded siderophores are transported actively through TonB- dependent transporters into the periplasm. The transport into the cytoplasm is usually mediated by ABC transporters in both Gram-positive and Gram-negative bacteria [36]. *S. aureus* for example, synthesizes two siderophores staphyloferrin A and staphyloferrin B, with the latter being a virulence factor for this bacterium [37]. In addition to these two siderophores, *S. aureus* synthesize also staphylopine, a broad spectrum opine type metallophore, that enables it to acquire several metal ions from the host other than iron such as cobalt nickel, and zinc [38] *P. aeruginosa*, on the other hand, own a metallophore encoding system resembling that of staphylopine and is responsible for synthesizing pseudopaline [35]. Compared to staphylopine, pseudopaline has lower metal ions specificity (narrow spectrum metallophores) [39].

The siderophore yersiniabactin and the nicotianamine-like yersinopine are the two metallophores secreted by *Y. pestis* [40,41]. This review highlights all the aspects related to yersiniabactin and yersinopine starting from synthesis along with genetic regulation, excretion, and uptake after sequestering metal ions. In addition, the siderophore aerobactin that was once produced in *Y. pestis* is also discussed herein.

## 2. Yersiniabactin

The transition metal iron is crucial for almost all pathogenic bacteria including *Y. pestis*. In the host metal ions’ scarce environment, bacteria developed various strategies to sequester iron. One of these strategies is the synthesis and secretion of siderophores which are low molecular weight metabolites specific for obtaining ferric ions. The highly pathogenic *Y. pestis* produces the siderophore yersiniabactin (Ybt), which has shown an important role in the acquisition of iron and murine pathogenicity [42]. Moreover, it was demonstrated that *Y. pestis* strains with mutations in genes responsible for Ybt biosynthesis or uptake caused an almost complete inability to generate fatal bubonic and pneumonic plague [43,44]. These findings prove that Ybt is associated with virulence besides acquiring iron.

### 2.1. Yersiniabactin Biosynthesis

**Figure 1:**
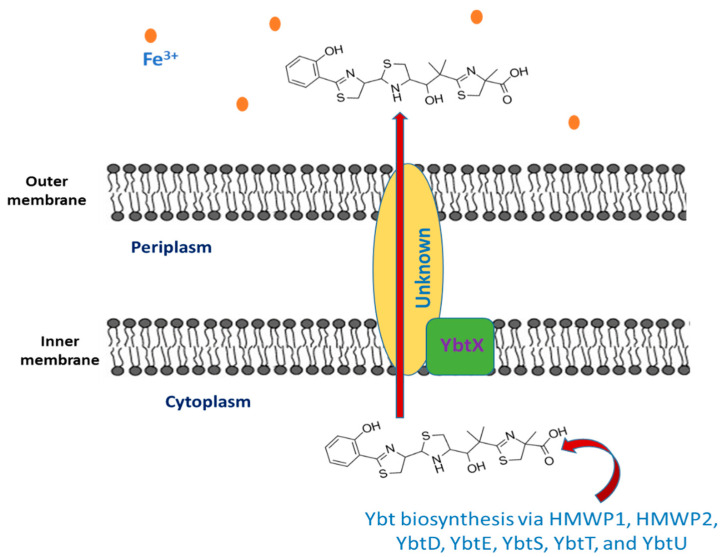
The export of yersiniabactin.

The siderophore Ybt is composed of thiazoline rings that are responsible for Fe^3+^ binding with a formation constant of 4 × 10^36^ [45]. The synthesis of Ybt is carried on by NRPS (nonribosomal peptide synthetase) mechanism combined with PKS (polyketide synthase). Ybt is constructed from salicylate, a linker malonyl group, three cysteines, and three methyl groups from S-adenosylmethionine [46]. These molecules yield a siderphore whose structure is composed of four rings (one salicylate, two thiazolines, and one thiazolidine) as shown in Figure 1.

Seven proteins (high molecular weight proteins 1 HMWP1, high molecular weight proteins 2 HMWP2, YbtD, YbtE, YbtS, YbtT, and YbtU) are involved in the synthesis of Ybt. The activated components of Ybt are bound to HMWP1 and HMWP2 via the coenzyme A moiety 4′-phosphopantetheine that is added by YbtD phosphopantetheinyl transferase to the carrier protein domains sites of these enzymes. Salicylate is synthesized from chorismate by YbtS, and then, it is activated through adenylation and transferred to HMWP2 by YbtE. Both HMWP1 and HMWP2 give the framing scaffold for the synthesis of Ybt. The NRPS enzymatic domains present in HMWP2 are responsible for cyclizing and condensing two cysteines, thus producing two thiazoline rings bonded to the salicylate component. The first four domains of the HMWP1 are the PKS module that performs the bis-methylation and the reduction in a malonyl linker moiety. The third molecule of cysteine that forms the final thiazoline ring is cyclized and condensed by two NRPS domains of HMWP1. As for the middle thiazoline ring, YbtU reduces it to thiazolidine. Then, the terminal HMWP1 thioesterase domain releases the synthesized siderophore. It is thought that the assumed type II thioesterase YbtT removes irregular molecules from the enzyme complex [47,48,49,50].

### 2.2. The Export of Ybt

Once the synthesis of Ybt is completed, it is exported outside the bacterial cell, but the mechanism underlying this process is still unknown. *Y. pestis* comprise YbtX, which is an inner membrane protein assigned to be a part of the Ybt efflux system because it resembles both AlcS and EntS exporters in alcaligin and enterobactin siderophores in *Bordetella* and *E. coli* respectively [51,52,53]. However, experiments showed that a *Y. pestis ybtX* mutant can secrete Ybt, uptake iron-loaded Ybt, and can grow under iron-scarce conditions as well [52]. The YbtX function is not fully understood, although it is involved in the secretion of Ybt, it is not essential in the process.

### 2.3. Iron Chelation by Ybt

As mentioned previously, Ybt contains a benzene ring, another thiazolidine ring, and two thiazoline rings. This structure provides five chiral centers (donor positions), enabling it to form a stable complex with trivalent ferric iron cations (Fe^3+^) with 1:1 complexes. The Ybt potential donor groups are: both the nitrogen found in the first thiazoline ring and the phenolic hydroxy group, either the nitrogen or the sulfur present in the thiazolidine moiety along with the aliphatic hydroxy group, as well as the carboxy group and the nitrogen of the terminal thiazoline ring [54].

### 2.4. The Intake of Iron-Loaded Ybt

**Figure 2:**
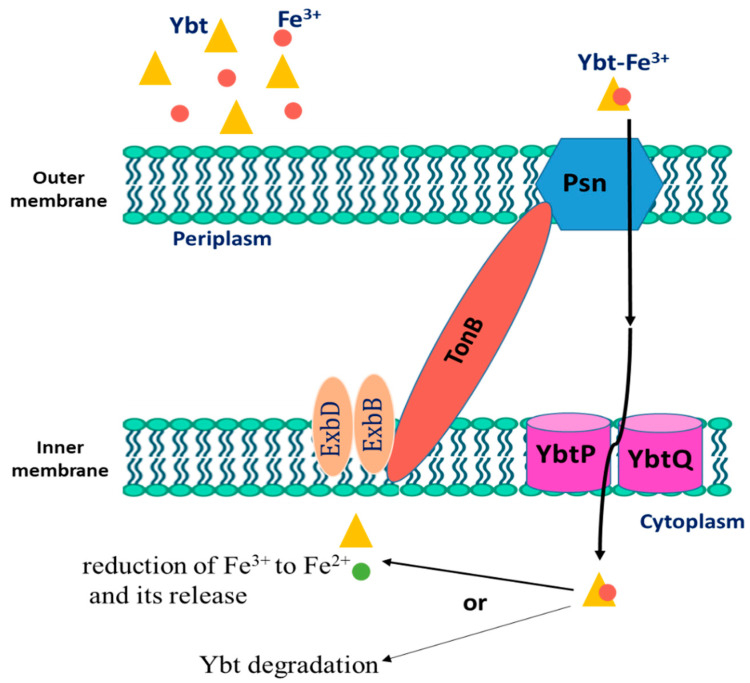
The uptake process of Fe^3+^-loaded Yersiniabactin (Ybt) by *Y. pestis*.

After Ybt chelates iron, the bacterium uptakes the loaded siderophore. The first step includes binding to the TonB-dependent receptor Psn, which is followed by outer membrane translocation. In Gram-negative bacteria, the function of TonB is associated with ExbB and ExbD, yet it is not ascertained in *Y. pestis*. Then, the iron-loaded Ybt is transported from the periplasm into the cytoplasm via the YbtP-YbtQ ABC transporter. The latter are similar inner membrane proteins with both ATPase and permease domains and are usually components of Type I secretion systems [55,56]. In the cytoplasm, Fe is then released in the cytoplasm from the siderophore by a reduction in ferric ions to ferrous ions or by Ybt degradation. The process of Ybt-Fe^3+^ uptake is demonstrated in Figure 2. Genes that encode either type of iron release mechanisms are not found within the high pathogenicity island (HPI) that is responsible for encoding most *ybt* genes. Noteworthily, *Y. pestis* mutant strains in either *psn* or *tonB* demonstrated both growth and iron uptake defects. Moreover, despite the presence of a second TonB-like gene (*hasB*), it is unable to be an alternative to TonB in taking iron through the Ybt system [57,58]. Moreover, strains with *ybtP* or *ybtQ* mutations have a similar phenotype *psn* mutant and showed reduced iron uptake and growth but without a notable reduction in Ybt production [55].

### 2.5. Genetic Regulation of Yersiniabactin (Ybt)

In *Y. pestis*, the *ybt* locus which is composed of four operons is responsible for *Ybt* biosynthesis, uptake, and possibly export. Out of the four operons, two are monocistronic, where one of them contains five genes encoding the biosynthetic enzymes, while the fourth operon genes encode the proteins involved in Ybt uptake and biosynthesis. Except for *ybtD*, *fur*, and the transport components (*tonB*, *exbB*, and *exbC*), all other genes involved in the function or regulation or function of the Ybt system are encoded within this locus [59]. The *ybt* locus is found within the high pathogenicity island (HPI) that was identified, for the first time, in the three *Yersinia* species causing pathogenesis in mammals. The *Y. pestis* HPI is found within the pigmentation (*pgm*) locus and comprises approximately one-third of it. The latter is an unstable wide area of the *Y. pestis* chromosome consisting of an HPI segment linked to a pigmentation segment [60]. The HPI is distributed widely among the *Enterobacteriaceae* family members. The IS*100* related to HPI of *Y. pestis* is not found in the other *Enterobacteriaceae*. The majority of the organisms having the HPI produce the proteins HMWP1 and HMWP2 in iron-scarce conditions and synthesize the siderophore Ybt [59].

As in other Gram-negative bacteria, *Y. pestis* produces iron uptake regulation protein (Fur), which is responsible for repressing the transcription of the promoters associated with a Fur binding sequence (FBS) once iron is in excess [60]. All of the four *ybt* operons that lie within the HPI possess FBSs promoters and are repressed via Fur approximately 12-fold (*psn*), 8-fold (*irp2-irp1-ybtUTE*), 11-fold (*ybtA*), and 55-fold (*ybtPQXS*) upon the growth with 10 μM iron compared to the growth in deferrated, defined media (PMH/PMH2) without additional iron [57,58,59,60,61]. *ybtD*, on the contrary, that is encoded outside the HPI and *pgm* locus is not Fur regulated.

### 2.6. The Role of Yersiniabactin in Overcoming Zinc Restriction

It was recently demonstrated that Ybt binds. thus enhancing the growth *Y. pestis* lacking ZnuABC transporter in zinc scarce medium. These findings suggest that Ybt may have a role in overcoming zinc nutritional immunity in addition to its role in iron sequestering. To test this assumption, Price et al. used an iron-mediated nutritional immunity defective mouse and demonstrated the contribution of Ybt to virulence independent of iron. In addition, they found that *Y. pestis* utilizes Ybt to compete for zinc with calprotectin. Furthermore, they discovered that in flea midgut, this pathogen relies on Ybt to survive zinc limitation [62]. Older studies showed that the *irp2* gene of *Y. pestis*, responsible for HMWP2 encoding, is needed for its growth in zinc-deficient medium for strains lacking ZnuABC [39]. Additionally, they showed that YbtX is required as well for the uptake of Ybt-dependent Zn^2+^ while both Psn and TonB, along with YbtPQ, are not required for zinc acquisition [63]. These results were further proved by *Bobrov* et al. who demonstrated that strains lacking both *ybtX* and *znu* genes were avirulent for bubonic and pneumonic plague in mice models at the time the virulence ability was conserved in strains mutant only for *ybtX* [64]. All these findings prove that Ybt has a role in the mechanism of zinc acquisition in *Y. pestis*, enabling it to overcome zinc limitation upon infecting both insects and mammals.

## 3. Yersinopine

**Figure 3:**
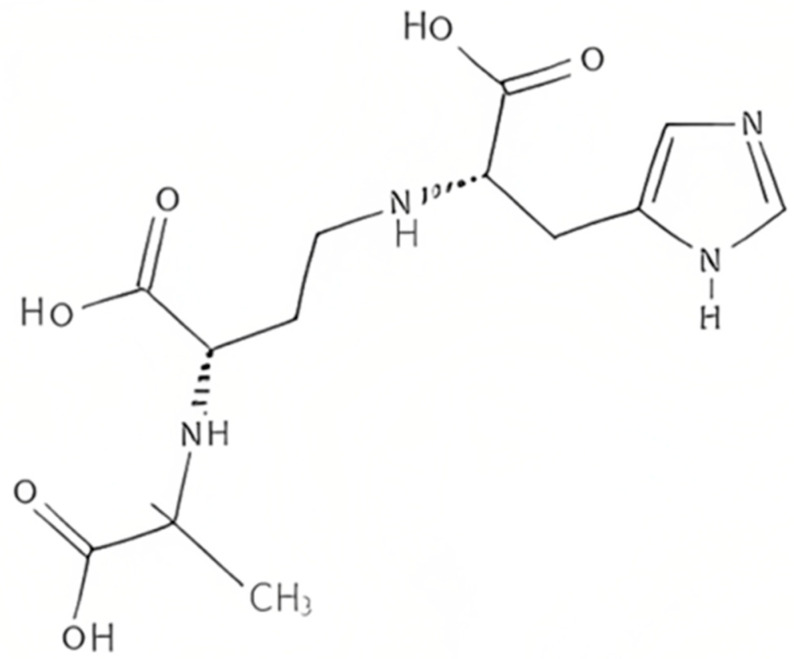
The structure of yersinopine.

Plants produce the metabolite nicotianamine, which is used in metal ions homeostasis such as zinc, iron, and nickel [65]. It is also synthesized by filamentous fungi [66] along with some mosses [67]. On the other hand, it was found that bacteria produce opine-type metallophores resembling nicotianamine, [68] in particular *Staphylococcus aureus*, *Pseudomonas aeruginosa*, and *Yersinia pestis.* These bacteria produce, respectively, staphylopine, pseudopaline, and yersinopine containing an imidazole ring and three carboxylic groups [69,70]. The structure of yersinopine is shown in Figure 3.

Two enzymes are involved in the biosynthesis of opine metallophores, the first is CntL, nicotianamine synthase (NAS), and the second is CntM, an opine dehydrogenase (ODH). The aminoalkyl transferase NAS which is *S*-adenosyl-L-methionine (SAM)-dependent forms a secondary amine between the aminobutyrate SAM component and amino acid [71]. In *P. aeruginosa*, the NAS uses _L_-histidine together with SAM to produce _L_-His-nicotianamine (_L_-HisNA), the substrate for *P. aeruginosa* ODH [31]. However, histidine racemase (CntK), which is a third enzyme produced by *S. aureus*, generates _D_-histidine used by the *S. aureus* NAS to produce _D_-His-nicotianamine (_D_-HisNA), the substrate for *Staphylococcus aureus* ODH [65]. Each one of these ODH binds to its HisNA substrate to perform a reductive condensation with an α-keto acid, which is followed by the reduction in NAD(P)H to generate the final opine metallophore. There exists a different substrate specificity between yersinopine dehydrogenase (YpODH) produced by *Y. pestis* compared to pseudopaline dehydrogenases. Pyruvate is selected by YpODH as its primary substrate, which is the case with *S. aureus* ODH while *P. aeruginosa* ODH is specific for α-ketoglutarate [68]. Such diversity in the opine-type metallophore is a result of possessing or lacking the *cntK* gene responsible for histidine racemase encoding [68].

It was confirmed that there is a strong link between bacterial infection and opine-type metallophores production in both *S. aureus* and *P. aeruginosa*. Starting with *S. aureus*, it was found that CntA, the receptor of staphylopine, is essential for optimal urease functioning as well as bacterium virulence. The deletion of this receptor causes a decrease in bacteremia and urinary tract infections in murine [72]. Concerning *P. aeruginosa*, the pseudopaline biosynthetic genes were found to be overexpressed in human burn wound infections [73]. Such overexpression allows surviving metal ions limitations during nutritional immunity. In addition, CntI, the exporter of pseudopaline, plays a crucial role in the growth and survival of *P. aeruginosa* in cystic fibrosis airway. Similarly, this exporter deletion leads to respiratory infection attenuation [74]. Moreover, the staphylopine exporter is important for *S. aureus* fitness in abscesses [75]. As for *Y. pestis*, there is no data currently available indicating a link between the production of yersinopine and virulence and the discovery of this metallophore is so far restricted to studies performed in vitro [76].

## 4. Aerobactin

The citrate-hydroxamate siderophore aerobactin is secreted by various pathogenic bacteria. Four biosynthetic enzymes (IucABCD) are encoded by the operon of aerobactin along with a transmembrane transporter (IutA) for its transport [77]. The genome of *Y. pestis* contains homologous genes to (*iutA*) and (*iucABCD*) in addition to the uptake system (*fhuCDB*) of ferric hydroxamate present in *Escherichia coli*. However, *iucA* is distorted by a frameshift mutation. Upon cloning the aerobactin region of *Y. pestis* to *E. coli*, the latter was unable to synthesize aerobactin; however, it was able to use this siderophore as an iron source [78]. Moreover, repairing the *iucA* frameshift mutation resulted in no aerobactin production in both *E. coli* and *Y. pestis*. Contrarily, inserting a plasmid with the genes *iucABCD-iutA* from *Shigella flexneri* into *Y. pestis* strain enabled it to synthesize and secrete aerobactin [78]. These results gave evidence concerning the ongoing *Y. pestis* genome fluidity. Such expansion and decay suggest that this pathogen went through a large genetic distortion, which gives an insight into the evolution ways that highly virulent pathogens undergo [79].

## 5. The Evolution of *Y. pestis*

*Y. pestis*, is considered a clone which emerged from the gastroenteric pathogen *Y. pseudotuberculosis* [80]. Approximately 97% similarity is shared at the chromosomal DNA level [80], and like other pathogenic Yersiniae, *Y. pestis* has the pCD1 plasmid, [81]. During evolution, *Y. pestis* acquired two additional plasmids (pMT1 and pPCP1) and a high pathogenicity island that consists of 32 chromosomal genes that are unique to *Y. pestis*. Some determinants encoded by the plasmids, pMT1, and pPCP1, facilitate *Y. pestis*-specific tissue invasion, survival in flea vectors, or possibly heavy growth in host blood [82]. Gene modification and loss are attributed to modifications of cellular structural or regulatory networks or elimination of activities no longer required for the *Y. pestis* new lifecycle [83]. For example, mutation or interruption of *yadA*, *inv*, and *ail* which encode adhesin or invasion attenuates the activities usually attributed to enteropathogenic virulence [84,85].

In contrast to the ancestor of *Y. pestis*, *Y. pseudotuberculosis*, a self-limiting gastroenteric pathogen, evolved to be a deadly pathogen occupying different niches [80]. *Y. pestis* only circulates within a narrow host range between rodent reservoir hosts and flea vectors in natural settings. The first challenge for *Y. pestis* survival in its lifecycle is sensing and adapting to temperature shifts while avoiding host innate immune cells during the early stage of infection and in host blood after release from innate immune cells, including macrophages. [86] This is problematic as *Y. pestis* develops into a systemic infection. During the complex lifestyle of *Y. pestis*, the intense or even life-threatening environmental changes are concomitant with a series of dynamic regulatory physiologic responses. However, *Y. pestis* physiology and pathogenesis, at the transcriptional and post-transcriptional level, is still far from understanding [87].

## 6. Conclusions

The pathogen *Yersinia pestis* is a very dangerous bacterium and is the causative agent of plague (septicemic, bubonic, and pneumonic). This Gram-negative bacterium is maintained in nature among rodents, where it is transmitted via a flea vector between them. It is also known for its potential to evade the host immune system and, thus, become resistant to phagocytosis. Metal ions such as iron and zinc are essential for bacterial cellular metabolism and virulence. During bacterial infection, the host restricts the metals’ availability to pathogens as a part of a defense mechanism known as nutritional immunity. Nevertheless, like many other bacteria, *Y. pestis* evolved several mechanisms to sequester metals from the host. One of these mechanisms is the production of siderophores, which are iron-chelating molecules. The *Y. pestis* siderophore is referred to as yersiniabactin (Ybt) and it is needed to survive iron nutritional immunity, thus causing a fatal infection. Moreover, Ybt has a role in evading zinc nutritional immunity as well during infection in mammals and in the colonization of *Y. pestis* in flea vectors. These facts make yersiniabactin, which is present in several pathogenic bacteria, essential for acquiring various metal ions during the process of overcoming nutritional immunity. Moreover, yersinopine is another opine-type metallophore produced by *Y. pestis* that resembles staphylopine and pseudopaline. It is worth mentioning that the plague caused by *Y. pestis* results in high death rates and due to the possible transmission between humans along with the absence of a vaccine approved by the FDA (Food and Drug Administration), this bacterium can be used as a biological weapon. Finally, based on what is mentioned above, it is crucial to understand the *Y. pestis* virulence and pathogenesis to develop powerful therapeutic approaches that protect against all kinds of *Y. pestis* infections threats either environmental or huma-made.

## Data Availability

No data were reported in this study.

## References

[1] Yang R., Atkinson S., Chen Z., Cui Y., Du Z., Han Y., Sebbane F., Slavin P., Song Y., Yan Y. (2023). *Yersinia pestis* and Plague: Some Knowns and Unknowns. Zoonoses.

[2] Eisen R.J., Bearden S.W., Wilder A.P., Montenieri J.A., Antolin M.F., Gage K.L. (2006). Early-phase transmission of *Yersinia pestis* by unblocked fleas as a mechanism explaining rapidly spreading plague epizootics. Proc. Natl. Acad. Sci. USA.

[3] Hinnebusch B.J., Jarrett C.O., Bland D.M. (2017). “Fleaing” the plague: Adaptations of *Yersinia pestis* to its insect vector that lead to transmission. Annu. Rev. Microbiol..

[4] Lathem W.W., Crosby S.D., Miller V.L., Goldman W.E. (2005). Progression of primary pneumonic plague: A mouse model of infection, pathology, and bacterial transcriptional activity. Proc. Natl. Acad. Sci. USA.

[5] Pechous R.D., Sivaraman V., Stasulli N.M., Goldman W.E. (2016). Pneumonic plague: The darker side of *Yersinia pestis*. Trends Microbiol..

[6] Dean K.R., Krauer F., Walløe L., Lingjærde O.C., Bramanti B., Stenseth N.C., Schmid B.V. (2018). Human ectoparasites and the spread of plague in Europe during the Second Pandemic. Proc. Natl. Acad. Sci. USA.

[7] Demeure C.E., Dussurget O., Fiol G.M., Le Guern A.-S., Savin C., Pizarro-Cerdá J. (2019). *Yersinia pestis* and plague: An updated view on evolution, virulence determinants, immune subversion, vaccination, and diagnostics. Genes Immun..

[8] Montminy S.W., Khan N., McGrath S., Walkowicz M.J., Sharp F., Conlon J.E., Fukase K., Kusumoto S., Sweet C., Miyake K. (2006). Virulence factors of *Yersinia pestis* are overcome by a strong lipopolysaccharide response. Nat. Immunol..

[9] Mordechai L., Eisenberg M., Newfield T.P., Izdebski A., Kay J.E., Poinar H. (2019). The Justinianic Plague: An inconsequential pandemic?. Proc. Natl. Acad. Sci. USA.

[10] Keller M., Spyrou M.A., Scheib C.L., Neumann G.U., Kröpelin A., Haas-Gebhard B., Päffgen B., Haberstroh J., Ribera I., Lacomba A. (2019). Ancient *Yersinia pestis* genomes from across Western Europe reveal early diversification during the First Pandemic (541–750). Proc. Natl. Acad. Sci. USA.

[11] Namouchi A., Guellil M., Kersten O., Hänsch S., Ottoni C., Schmid B.V., Pacciani E., Quaglia L., Vermunt M., Bauer E.L. (2018). Integrative approach using *Yersinia pestis* genomes to revisit the historical landscape of plague during the Medieval Period. Proc. Natl. Acad. Sci. USA.

[12] Gonzalez R.J., Miller V.L. (2016). A deadly path: Bacterial spread during bubonic plague. Trends Microbiol..

[13] Arbaji A., Kharabsheh S., Al-Azab S., Al-Kayed M., Amr Z.S., Abu Baker M., Chu M.C. (2005). A 12-case outbreak of pharyngeal plague following the consumption of camel meat. Ann. Trop. Med. Parasitol..

[14] Randremanana R., Andrianaivoarimanana V., Nikolay B., Ramasindrazana B., Paireau J., ten Bosch Q.A., Rakotondramanga J.M., Rahajandraibe S., Rahelinirina S., Rakotomanana F. (2019). Epidemiological characteristics of an urban plague epidemic in Madagascar, August–November, 2017: An outbreak report. Lancet Infect. Dis..

[15] Guiyoule A., Gerbaud G., Buchrieser C., Galimand M., Rahalison L., Chanteau S., Courvalin P., Carniel E. (2001). Transferable plasmid-mediated resistance to streptomycin in a clinical isolate of *Yersinia pestis*. Emerg. Infect. Dis..

[16] Galimand M., Carniel E., Courvalin P. (2006). Resistance of *Yersinia pestis* to antimicrobial agents. Antimicrob. Agents Chemother..

[17] Cornelius C.A., Quenee L.E., Overheim K.A., Koster F., Brasel T.L., Elli D., Ciletti N.A., Schneewind O. (2008). Immunization with recombinant V10 protects cynomolgus macaques from lethal pneumonic plague. Infect. Immun..

[18] Becker K.W., Skaar E.P. (2014). Metal limitation and toxicity at the interface between host and pathogen. FEMS Microbiol. Rev..

[19] Lopez C.A., Skaar E.P. (2018). The impact of dietary transition metals on host-bacterial interactions. Cell Host Microbe.

[20] Skaar E.P., Raffatellu M. (2015). Metals in infectious diseases and nutritional immunity. Metallomics.

[21] Lonergan Z.R., Skaar E.P. (2019). Nutrient zinc at the host-pathogen interface. Trends Biochem. Sci..

[22] Palmer L.D., Skaar E.P. (2016). Transition Metals and Virulence in Bacteria. Annu. Rev. Genet..

[23] Brinkmann V., Zychlinsky A. (2012). Neutrophil extracellular traps: Is immunity the second function of chromatin?. J. Cell Biol..

[24] Sohnle P.G., Hunter M.J., Hahn B., Chazin W.J. (2000). Zinc-reversible antimicrobial activity of recombinant calprotectin (migration inhibitory factor-related proteins 8 and 14). J. Infect. Dis..

[25] Kelliher J.L., Kehl-Fie T.E. (2016). Competition for manganese at the host-pathogen interface. Prog. Mol. Biol. Transl. Sci..

[26] Damo S.M., Kehl-Fie T.E., Sugitani N., Holt M.E., Rathi S., Murphy W.J., Zhang Y., Betz C., Hench L., Fritz G. (2013). Molecular basis for manganese sequestration by calprotectin and roles in the innate immune response to invading bacterial pathogens. Proc. Natl. Acad. Sci. USA.

[27] Kehl-Fie T.E., Chitayat S., Hood M.I., Damo S., Restrepo N., Garcia C., Munro K.A., Chazin W.J., Skaar E.P. (2011). Nutrient metal sequestration by calprotectin inhibits bacterial superoxide defense, enhancing neutrophil killing of *Staphylococcus aureus*. Cell Host Microbe.

[28] Liu J.Z., Jellbauer S., Poe A.J., Ton V., Pesciaroli M., Kehl-Fie T.E., Restrepo N.A., Hosking M.P., Edwards R.A., Battistoni A. (2012). Zinc sequestration by the neutrophil protein calprotectin enhances Salmonella growth in the inflamed gut. Cell Host Microbe.

[29] Aisen P., Enns C., Wessling-Resnick M. (2001). Chemistry and biology of eukaryotic iron metabolism. Int. J. Biochem. Cell Biol..

[30] Braun V., Hantke K. (2011). Recent insights into iron import by bacteria. Curr. Opin. Chem. Biol..

[31] Andrews S.C., Robinson A.K., Rodríguez Quiñones F. (2003). Bacterial iron homeostasis. FEMS Microbiol. Rev..

[32] Masson P.L., Heremans J.F., Schonne E. (1969). Lactoferrin, an iron-binding protein in neutrophilic leukocytes. J. Exp. Med..

[33] Rakin A., Schneider L., Podladchikova O. (2012). Hunger for iron: The alternative siderophore iron scavenging systems in highly virulent *Yersinia*. Front. Cell. Infect. Microbiol..

[34] Schalk I.J., Hannauer M., Braud A. (2011). New roles for bacterial siderophores in metal transport and tolerance. Environ. Microbiol..

[35] Lhospice S., Gomez N.O., Ouerdane L., Brutesco C., Ghssein G., Hajjar C., Liratni A., Wang S., Richaud P., Bleves S. (2017). Pseudomonas aeruginosa zinc uptake in chelating environment is primarily mediated by the metallophore pseudopaline. Sci. Rep..

[36] Cornelissen C.N., Hollander A. (2011). TonB-Dependent Transporters Expressed by *Neisseria gonorrhoeae*. Front. Microbiol..

[37] Dale S.E., Doherty-Kirby A., Lajoie G., Heinrichs D.E. (2004). Role of siderophore biosynthesis in virulence of *Staphylococcus aureus*: Identification and characterization of genes involved in production of a siderophore. Infect. Immun..

[38] Ghssein G., Matar S.F. (2018). Chelating Mechanisms of Transition Metals by Bacterial Metallophores “Pseudopaline and Staphylopine”: A Quantum Chemical Assessment. Computation.

[39] Mastropasqua M.C., D′Orazio M., Cerasi M., Pacello F., Gismondi A., Canini A., Canuti L., Consalvo A., Ciavardelli D., Chirullo B. (2017). Growth of Pseudomonas aeruginosa in zinc poor environments is promoted by a nicotianamine-related metallophore. Mol. Microbiol..

[40] Perry R.D., Balbo P.B., Jones H.A., Fetherston J.D., DeMoll E. (1999). Yersiniabactin from *Yersinia pestis*: Biochemical characterization of the siderophore and its role in iron transport and regulation. Microbiology.

[41] McFarlane J.S., Davis C.L., Lamb A.L. (2018). Staphylopine, pseudopaline, and yersinopine dehydrogenases: A structural and kinetic analysis of a new functional class of opine dehydrogenase. J. Biol. Chem..

[42] Rakin A., Saken E., Harmsen D., Heesemann J. (1994). The pesticin receptor of *Yersinia enterocolitica*: A novel virulence factor with dual function. Mol. Microbiol..

[43] Fetherston J.D., Kirillina O., Bobrov A.G., Paulley J.T., Perry R.D. (2010). The yersiniabactin transport system is critical for the pathogenesis of bubonic and pneumonic plague. Infect. Immun..

[44] Lee-Lewis H., Anderson D.M. (2009). Absence of inflammation and pneumonia during infection with nonpigmented *Yersinia pestis* reveals a new role for the pgm locus in pathogenesis. Infect. Immun..

[45] Perry R.D., Fetherston J.D. (2011). Yersiniabactin iron uptake: Mechanisms and role in *Yersinia pestis* pathogenesis. Microbes Infect..

[46] Chambers C.E., McIntyre D.D., Mouck M., Sokol P.A. (1996). Physical and structural characterization of yersiniophore, a siderophore produced by clinical isolates of Yersinia enterocolitica. Biometals.

[47] Bearden S.W., Fetherston J.D., Perry R.D. (1997). Genetic organization of the yersiniabactin biosynthetic region and construction of avirulent mutants in *Yersinia pestis*. Infect. Immun..

[48] Gehring A.M., DeMoll E., Fetherston J.D., Mori I., Mayhew G.F., Blattner F.R., Walsh C.T., Perry R.D. (1998). Iron acquisition in plague: Modular logic in enzymatic biogenesis of yersiniabactin by *Yersinia pestis*. Chem. Biol..

[49] Bobrov A.G., Geoffroy V.A., Perry R.D. (2002). Yersiniabactin production requires the thioesterase domain of HMWP2 and YbtD, a putative phosphopantetheinylate transferase. Infect. Immun..

[50] Geoffroy V.A., Fetherston J.D., Perry R.D. (2000). *Yersinia pestis* YbtU and YbtT are involved in synthesis of the siderophore yersiniabactin but have different effects on regulation. Infect. Immun..

[51] Miller M.C., Fetherston J.D., Pickett C.L., Bobrov A.G., Weaver R.H., DeMoll E., Perry R.D. (2010). Reduced synthesis of the Ybt siderophore or production of aberrant Ybt-like molecules activates transcription of yersiniabactin genes in *Yersinia pestis*. Microbiology.

[52] Furrer J.L., Sanders D.N., Hook-Barnard I.G., McIntosh M.A. (2002). Export of the siderophore enterobactin in *Escherichia coli*: Involvement of a 43 kDa membrane exporter. Mol. Microbiol..

[53] Brickman T.J., Armstrong S.K. (2005). *Bordetella* AlcS transporter functions in alcaligin siderophore export and is central to inducer sensing in positive regulation of alcaligin system gene expression. J. Bacteriol..

[54] Drechsel H., Stephan H., Lotz R., Haag H., Zähner H., Hantke K., Jung G. (1995). Structure elucidation of yersiniabactin, a siderophore from highly virulent Yersinia strains. Liebigs Ann..

[55] Fetherston J.D., Bertolino V.J., Perry R.D. (1999). YbtP and YbtQ: Two ABC transporters required for iron uptake in *Yersinia pestis*. Mol. Microbiol..

[56] Buchanan S.K. (2001). Type I secretion and multidrug efflux: Transport through the TolC channel-tunnel. Trends Biochem. Sci..

[57] Fetherston J.D., Lillard J.W., Perry R.D. (1995). Analysis of the pesticin receptor from *Yersinia pestis*: Role in iron-deficient growth and possible regulation by its siderophore. J. Bacteriol..

[58] Perry R.D., Shah J., Bearden S.W., Thompson J.M., Fetherston J.D. (2003). *Yersinia pestis* TonB: Role in iron, heme and hemoprotein utilization. Infect. Immun..

[59] Lesic B., Carniel E., Carniel E., Hinnebusch B.J. (2004). Yersinia Molecular and Cellular Biology. Horizon Bioscience.

[60] Leal-Balbino T.C., Leal N.C., Nascimento M.G.M.D., Oliveira M.B.M.D., Balbino V.D.Q., Almeida A.M.P.D. (2006). The pgm locus and pigmentation phenotype in *Yersinia pestis*. Genet. Mol. Biol..

[61] Gao H., Zhou D., Li Y., Guo Z., Han Y., Song Y., Zhai J., Du Z., Wang X., Lu J. (2008). The ironresponsive Fur regulon in *Yersinia pestis*. J. Bacteriol..

[62] Price S.L., Vadyvaloo V., DeMarco J.K., Brady A., Gray P.A., Kehl-Fie T.E., Garneau-Tsodikova S., Perry R.D., Lawrenz M.B. (2021). Yersiniabactin contributes to overcoming zinc restriction during *Yersinia pestis* infection of mammalian and insect hosts. Proc. Natl. Acad. Sci. USA.

[63] Bobrov A.G., Kirillina O., Fetherston J.D., Miller M.C., Burlison J.A., Perry R.D. (2014). The *Yersinia pestis* siderophore, yersiniabactin, and the ZnuABC system both contribute to zinc acquisition and the development of lethal septicaemic plague in mice. Mol. Microbiol..

[64] Bobrov A.G., Kirillina O., Fosso M.Y., Fetherston J.D., Miller M.C., VanCleave T.T., Burlison J.A., Arnold W.K., Lawrenz M.B., Garneau-Tsodikova S. (2017). Zinc transporters YbtX and ZnuABC are required for the virulence of *Yersinia pestis* in bubonic and pneumonic plague in mice. Metallomics.

[65] Reichman S.M., Parker D.R. (2002). Revisiting the metal-binding chemistry of nicotianamine and 2′-deoxymugineic acid. Implications for iron nutrition in strategy II plants. Plant Physiol..

[66] Trampczynska A., Böttcher C., Clemens S. (2006). The transition metal chelator nicotianamine is synthesized by filamentous fungi. FEBS Lett..

[67] Burkhead J.L., Gogolin Reynolds K.A., Abdel-Ghany S.E., Cohu C.M., Pilon M. (2009). Copper homeostasis. New Phytol..

[68] Ghssein G., Ezzeddine Z. (2022). The Key Element Role of Metallophores in the Pathogenicity and Virulence of *Staphylococcus aureus*: A Review. Biology.

[69] Ghssein G., Ezzeddine Z. (2022). A Review of *Pseudomonas aeruginosa* Metallophores: Pyoverdine, Pyochelin and Pseudopaline. Biology.

[70] Ghssein G., Brutesco C., Ouerdane L., Fojcik C., Izaute A., Wang S., Hajjar C., Lobinski R., Lemaire D., Richaud P. (2016). Biosynthesis of a broad-spectrum nicotianamine-like metallophore in *Staphylococcus aureus*. Science.

[71] McFarlane J.S., Lamb A.L. (2017). Biosynthesis of an opine metallophore by *Pseudomonas aeruginosa*. Biochemistry.

[72] Laffont C., Brutesco C., Hajjar C., Cullia G., Fanelli R., Ouerdane L., Cavelier F., Arnoux P. (2019). Simple rules govern the diversity of bacterial nicotianamine-like metallophores. Biochem. J..

[73] Remy L., Carrière M., Derré-Bobillot A., Martini C., Sanguinetti M., Borezée-Durant E. (2013). The *Staphylococcus aureus* Opp1 ABC transporter imports nickel and cobalt in zinc depleted conditions and contributes to virulence: Nickel and cobalt uptake in *Staphylococcus aureus*. Mol. Microbiol..

[74] Bielecki P., Puchałka J., Wos-Oxley M.L., Loessner H., Glik J., Kawecki M., Nowak M., Tümmler B., Weiss S., dos Santos V.A.P.M. (2011). In-Vivo Expression Profiling of *Pseudomonas aeruginosa* Infections Reveals Niche-Specific and Strain-Independent Transcriptional Programs. PLoS ONE.

[75] Gi M., Lee K.M., Kim S.C., Yoon J.H., Yoon S.S., Choi J.Y. (2015). A novel siderophore system is essential for the growth of *Pseudomonas aeruginosa* in airway mucus. Sci. Rep..

[76] Ding Y., Fu Y., Lee J.C., Hooper D.C. (2012). *Staphylococcus aureus* NorD, a Putative Efflux Pump Coregulated with the Opp1 Oligopeptide Permease, Contributes Selectively to Fitness In Vivo. J. Bacteriol..

[77] Li C., Pan D., Li M., Wang Y., Song L., Yu D., Zuo Y., Wang K., Liu Y., Wei Z. (2021). Aerobactin-Mediated Iron Acquisition Enhances Biofilm Formation, Oxidative Stress Resistance, and Virulence of *Yersinia pseudotuberculosis*. Front. Microbiol..

[78] Forman S., Nagiec M.J., Abney J., Perry R.D., Fetherston J.D. (2007). Analysis of the aerobactin and ferric hydroxamate uptake systems of *Yersinia pestis*. Microbiology.

[79] Parkhill J., Wren B.W., Thomson N.R., Titball R.W., Holden M.T.G., Prentice M.B., Sebaihia M., James K.D., Churcher C., Mungall K.L. (2001). Genome sequence of *Yersinia pestis*, the causative agent of plague. Nature.

[80] Achtman M., Zurth K., Morelli G., Torrea G., Guiyoule A., Carniel E. (1999). *Yersinia pestis*, the cause of plague, is a recently emerged clone of *Yersinia pseudotuberculosis*. Proc. Natl. Acad. Sci. USA.

[81] Cornelis G.R., Boland A., Boyd A.P., Geuijen C., Iriarte M., Neyt C., Sory M.P., Stainier I. (1998). The virulence plasmid of Yersinia, an antihost genome. Microbiol. Mol. Biol. Rev..

[82] Lathem W.W., Price P.A., Miller V.L., Goldman W.E. (2007). A plasminogen-activating protease specifically controls the development of primary pneumonic plague. Science.

[83] Sebbane F., Jarrett C.O., Gardner D., Long D., Hinnebusch B.J. (2006). Role of the *Yersinia pestis* plasminogen activator in the incidence of distinct septicemic and bubonic forms of flea-borne plague. Proc. Natl. Acad. Sci. USA.

[84] Mcnally A., Thomson N.R., Reuter S., Wren B.W. (2016). ‘Add, stir and reduce’: *Yersinia* spp. as model bacteria for pathogen evolution. Nat. Rev. Microbiol..

[85] Chain P.S., Hu P., Malfatti S.A., Radnedge L., Larimer F., Vergez L.M., Worsham P., Chu M.C., Andersen G.L. (2006). Complete genome sequence of *Yersinia pestis* strains Antiqua and Nepal516: Evidence of gene reduction in an emerging pathogen. J. Bacteriol..

[86] Zhao Y., Wang T., Liu Z., Ke Y., Li R., Chen H., You Y., Wu G., Cao S., Du Z. (2023). Single-cell transcriptomics of immune cells in lymph nodes reveals their composition and alterations in functional dynamics during the early stages of bubonic plague. Sci. China Life Sci..

[87] Heroven A.K., Dersch P. (2014). Coregulation of host-adapted metabolism and virulence by pathogenic yersiniae. Front. Cell. Infect. Microbiol..

